# A new monoclinic polymorph of dichlorido­tetra­kis(dimethyl sulfoxide)­ruthenium(II)

**DOI:** 10.1107/S160053680801996X

**Published:** 2008-07-12

**Authors:** Gergana Georgieva, Galina Gencheva, Boris Lubomirov Shivachev, Rosica Petrova Nikolova

**Affiliations:** a Sofia University, Faculty of Chemistry, 1 James Bourchier Boulevard, 1164 Sofia, Bulgaria; b Department of Structural Biology, University of Pittsburgh School of Medicine, 3501 5th Avenue, Pittsburgh, PA 15260, USA; c Department of Advanced Materials Science and Engineering, Faculty of Engineering, Yamaguchi University, 2-16-1 Tokiwadai, Ube 755-8611, Japan

## Abstract

The title compound, *cis*,*fac*-dichloridotetra­kis(dimethyl sulfoxide)-κ^3^ *S*,κ*O*-ruthenium(II), [RuCl_2_(C_2_H_6_OS)_4_], was obtained from newly synthesized ruthenium complexes of 3-amino-2-chloro­pyridine. The Ru atom has a distorted octa­hedral coordination with two *cis*-oriented chloride ligands and four dimethyl sulfoxide ligands. Three of the sulfoxide ligands are *S*-bonded in a *fac* configuration, while the fourth is *O*-bonded. The title compound represents a new, and fourth, polymorph of the complex. Two other monoclinic forms and an ortho­rhom­bic modification have been reported previously.

## Related literature

For the geometric parameters and crystal structures of related polymorphs, see: Alessio *et al.* (1988 ▶); Attia & Calligaris (1987 ▶); Galanski *et al.* (2003 ▶); Mercer & Trotter (1975 ▶); Pigge *et al.* (2005 ▶); Srivastava & Fronczek (2003 ▶).
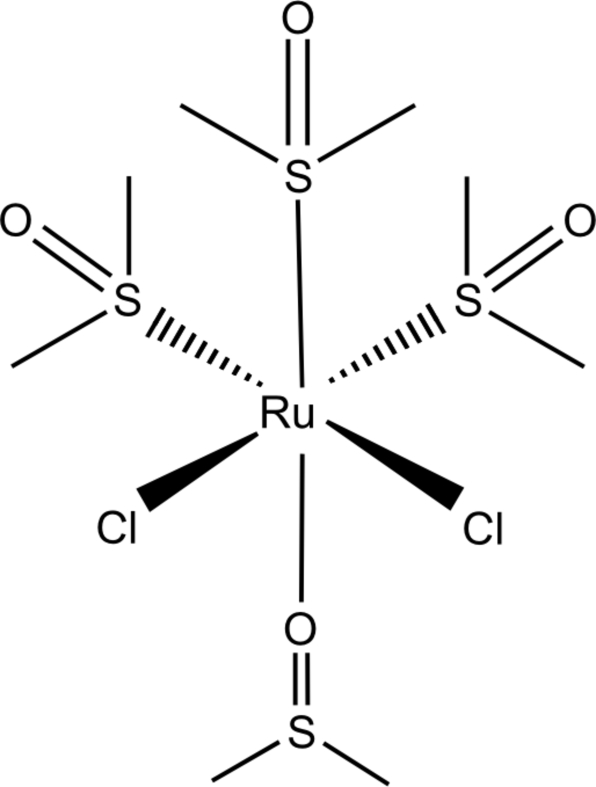


## Experimental

### 

#### Crystal data

[RuCl_2_(C_2_H_6_OS)_4_]
*M* *_r_* = 484.48
Monoclinic, 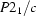

*a* = 10.1479 (3) Å
*b* = 10.4626 (3) Å
*c* = 18.4280 (4) Å
β = 99.795 (14)°
*V* = 1928.04 (12) Å^3^
*Z* = 4
Mo *K*α radiation
μ = 1.53 mm^−1^
*T* = 290 (2) K
0.29 × 0.26 × 0.25 mm

#### Data collection

Enraf–Nonius CAD-4 diffractometer
Absorption correction: none
7705 measured reflections
3777 independent reflections
2953 reflections with *I* > 2σ(*I*)
*R*_int_ = 0.045
3 standard reflections frequency: 120 min intensity decay: 1%

#### Refinement

*R*[*F* ^2^ > 2σ(*F* ^2^)] = 0.034
*wR*(*F* ^2^) = 0.077
*S* = 1.03
3777 reflections
172 parameters
H-atom parameters constrained
Δρ_max_ = 0.36 e Å^−3^
Δρ_min_ = −0.64 e Å^−3^

### 

Data collection: *CAD-4 EXPRESS* (Enraf–Nonius, 1994 ▶); cell refinement: *CAD-4 EXPRESS*; data reduction: *XCAD4* (Harms & Wocadlo, 1995 ▶); program(s) used to solve structure: *SHELXS97* (Sheldrick, 2008 ▶); program(s) used to refine structure: *SHELXL97* (Sheldrick, 2008 ▶); molecular graphics: *ORTEP-3 for Windows* (Farrugia, 1997 ▶) and *Mercury* (Macrae *et al.*, 2006 ▶); software used to prepare material for publication: *WinGX* (Farrugia, 1999 ▶).

## Supplementary Material

Crystal structure: contains datablocks I, global. DOI: 10.1107/S160053680801996X/fj2125sup1.cif

Structure factors: contains datablocks I. DOI: 10.1107/S160053680801996X/fj2125Isup2.hkl

Additional supplementary materials:  crystallographic information; 3D view; checkCIF report

## Figures and Tables

**Fig. 1.**
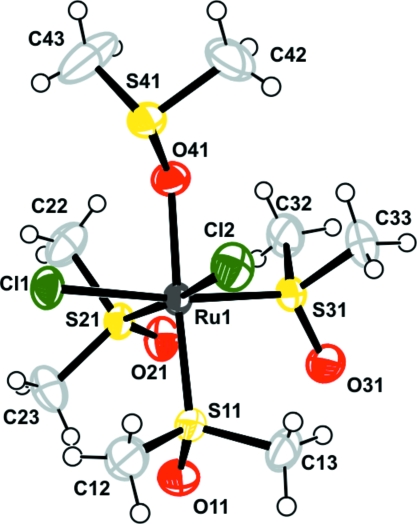
View of the structure and the atom-numbering scheme of (I) showing 50% probability displacement ellipsoids. H atoms are shown as small spheres of arbitrary radii.

**Fig. 2.**
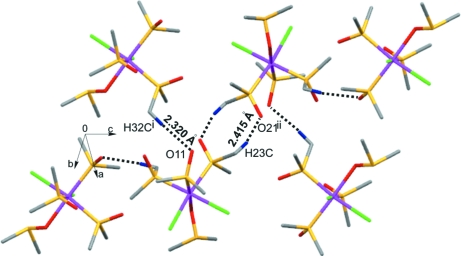
A view of the molecular packing in (I). Short contacts are represented by dotted lines. All H atoms except those involved in the short contacts have been omitted. [Symmetry codes: (i) -x, y - 1/2,1/2 - z; (ii) -x1, -y, 1 - z].

**Fig. 3.**
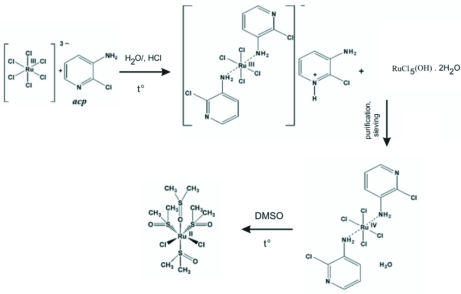
The preparation of the title compound.

## supplementary crystallographic information

### Comment

In the field of medicinal chemistry ruthenium complexes have gained considerable attention as non-platinum anticancer agents (Galanski *et al.*, 2003). In order to obtain new complexes with potential antitumor properties we studied the reactions of Ru(III) and 3-amino-2-chloropyridine (*acp*) under different conditions. We found that along with the complexation reaction a redox process also takes places leading to the formation of ruthenium complexes with the studied bidentate N-ligand in different oxidation states. The reported titled compound is a side product of this reaction.

The new polymorph modification of the title compound (Fig. 1) was obtained studying the substitution reaction of the *acp*-ligands from the inner coordination sphere of *trans*-[Ru(IV)Cl_4_(*acp*)_2_] in a hot DMSO solution (120–130 °C) (Fig. 3). The resulting orange crystals were determined by crystal structure analysis to be *cis*-RuCl_2_(DMSO)_4_ (Fig. 1). Two monoclinic (Mercer & Trotter, 1975; Alessio *et al.*, 1988; Pigge *et al.*, 2005) and one orthorhombic (Attia & Calligaris, 1987; Srivastava & Fronczek, 2003) modifications have been previously reported. The compound reported here is also monoclinic with similar type of ruthenium coordination but different three-dimensional arrangement of the structural units. Of the four DMSO ligands, three are S-coordinated, with Ru–S distances of 2.240 (2), 2.264 (2) and 2.278 (2) Å. As already reported, the Ru–S distance *trans* to the O-bonded DMSO (2.240 (2) Å) is significantly shortened, being more than 0.02 Å shorter than the other two.

The 0.05 Å longer S—O distance in the O-bonded DMSO ligand, is indicative for the weakened double-bond character compared to the other three DMSO ligands. As it was mentioned above the only difference between the polymorphs is the three-dimensional arrangement of the structural units. The difference is easily recognized if the number and arrangement of short O···H contacts between the neighboring entities is considered.

We calculated the O···H contacts with distances shorter than the sum of Van der Waals radii (*e.g.* less than 2.5 Å) for all previously reported polymorphs and for the second title compound.

In the case of the orthorhombic structure there is only one such contact of 2.296 Å between O3(S-bonded DMSO) and H6(O-bonded DMSO) (Srivastava & Fronczek, 2003); for the same polymorph refined earlier by Attia & Calligaris, 1987, the obtained distance is 2.391 Å and occurs between O3 and H5 atoms). The structural units connected by that short contact are chain-like arranged along *c* axis. Similar arrangement of the structural units is observed in the monoclinic modifications with *β* angle close to 90° (Mercer & Trotter, 1975; Pigge *et al.*, 2005). The obtained O···H distances are 2.375 and 2.443 Å for the structures reported in 1995 and 2005 respectively. In the second monoclinic polymorph with *β* angle of 116.8° there are no O···H contacts below 2.5 Å, the shortest one is of 2.546 Å and occurs between O2 and H7 (both oxygen and hydrogen atoms belong to S-bonded DMSO molecules). Nevertheless, the arrangement of the units connected through the "shortest" contact is chain-like and is analogous to the one in the other polymorphs.

In the structure of (I) there are two O···H contacts shorter than 2.5 Å, both between atoms belonging to only S-bonded DMSO molecules. As it is shown in Fig. 2 the structural units are connected by the described contacts in two directions in a layer–like arrangement differing from the chain-like one found in earlier reported polymorphs.

### Experimental

Compound (I) was obtained from a 1*M* HCl hydrochloric-water solution of RuCl_3_.H_2_O (0.3 mmol, 0.0633 g) and 3-amino-2-chloropyridine (2.4 mmol, 0.3085 g). The mixture was left for 48 h at ambient temperature. The obtainedSome H[RuCl_5_OH].2H_2_O together with the main product *trans*-[Ru(III)Cl_4_(acp)_2_][H*acp*] were separated by sieving. A sample of the purified *trans*-[Ru(III)Cl_4_(acp)_2_][H*acp*] (0.5 g, 0.96 mmol) was dissolved in 2 ml DMSO and the solution was heated at (135±5 °C) for 30 min. The solution was then cooled to room temperature. Clear yellow-orange crystals of *cis*,*fac*-[Ru(II)Cl_2_(C_2_H_6_OS)_4_] were obtained several days later by slow diffusion of acetone and a few drops of diethyl ether into the solution.

### Refinement

The methyl H atoms were placed in idealized positions (*C*—H_methyl_ = 0.96 Å). All H atoms were constrained to ride on their parent atoms, with *U*_iso_(H) = 1.5*U*_eq_(C_methyl_).

### Crystal data

**Table:**

[RuCl_2_(C_2_H_6_OS)_4_]	*F*(000) = 984
*M**_r_* = 484.48	*D*_x_ = 1.669 Mg m^−^^3^
Monoclinic, *P*2_1_/*c*	Melting point: not measured K
Hall symbol: -P 2ybc	Mo *K*α radiation, λ = 0.71073 Å
*a* = 10.1479 (3) Å	Cell parameters from 22 reflections
*b* = 10.4626 (3) Å	θ = 18.2–19.2°
*c* = 18.4280 (4) Å	µ = 1.53 mm^−^^1^
β = 99.795 (14)°	*T* = 290 K
*V* = 1928.04 (12) Å^3^	Prism, orange
*Z* = 4	0.29 × 0.26 × 0.25 mm

### Data collection

**Table:**

Enraf–Nonius CAD-4 diffractometer	*R*_int_ = 0.045
Radiation source: Enraf Nonius FR590	θ_max_ = 26.0°, θ_min_ = 2.0°
graphite	*h* = 0→12
non–profiled ω/2θ scans	*k* = −12→12
7705 measured reflections	*l* = −22→22
3777 independent reflections	3 standard reflections every 120 min
2953 reflections with *I* > 2σ(*I*)	intensity decay: 1%

### Refinement

**Table:**

Refinement on *F*^2^	Primary atom site location: structure-invariant direct methods
Least-squares matrix: full	Secondary atom site location: difference Fourier map
*R*[*F*^2^ > 2σ(*F*^2^)] = 0.034	Hydrogen site location: inferred from neighbouring sites
*wR*(*F*^2^) = 0.077	H-atom parameters constrained
*S* = 1.03	*w* = 1/[σ^2^(*F*_o_^2^) + (0.0294*P*)^2^] where *P* = (*F*_o_^2^ + 2*F*_c_^2^)/3
3777 reflections	(Δ/σ)_max_ < 0.001
172 parameters	Δρ_max_ = 0.36 e Å^−^^3^
0 restraints	Δρ_min_ = −0.64 e Å^−^^3^

### Special details

**Table:**

Geometry. All e.s.d.'s (except the e.s.d. in the dihedral angle between two l.s. planes) are estimated using the full covariance matrix. The cell e.s.d.'s are taken into account individually in the estimation of e.s.d.'s in distances, angles and torsion angles; correlations between e.s.d.'s in cell parameters are only used when they are defined by crystal symmetry. An approximate (isotropic) treatment of cell e.s.d.'s is used for estimating e.s.d.'s involving l.s. planes.
Refinement. Refinement of *F*^2^ against ALL reflections. The weighted *R*-factor *wR* and goodness of fit *S* are based on *F*^2^, conventional *R*-factors *R* are based on *F*, with *F* set to zero for negative *F*^2^. The threshold expression of *F*^2^ > σ(*F*^2^) is used only for calculating *R*-factors(gt) *etc*. and is not relevant to the choice of reflections for refinement. *R*-factors based on *F*^2^ are statistically about twice as large as those based on *F*, and *R*- factors based on ALL data will be even larger.

### Fractional atomic coordinates and isotropic or equivalent isotropic displacement parameters (Å^2^)

**Table:**

	*x*	*y*	*z*	*U*_iso_*/*U*_eq_	
O11	0.2531 (3)	−0.0625 (3)	0.32395 (16)	0.0530 (8)	
O21	0.0063 (3)	0.0806 (3)	0.41116 (15)	0.0465 (7)	
O31	0.0400 (3)	0.1627 (3)	0.23279 (15)	0.0543 (8)	
O41	0.2122 (3)	0.4088 (2)	0.42297 (14)	0.0400 (6)	
C12	0.5002 (4)	0.0204 (5)	0.3510 (2)	0.0603 (13)	
H12A	0.5531	0.0958	0.3487	0.09*	
H12B	0.5283	−0.0448	0.3203	0.09*	
H12C	0.5116	−0.0095	0.4009	0.09*	
C13	0.3327 (5)	0.0822 (4)	0.2244 (2)	0.0566 (13)	
H13A	0.2448	0.1037	0.1992	0.085*	
H13B	0.3627	0.0057	0.2036	0.085*	
H13C	0.3931	0.1509	0.2192	0.085*	
C22	0.1268 (5)	0.2105 (4)	0.5245 (2)	0.0557 (12)	
H22A	0.0838	0.2906	0.5104	0.084*	
H22B	0.214	0.226	0.5527	0.084*	
H22C	0.0742	0.163	0.5538	0.084*	
C23	0.2246 (4)	−0.0186 (4)	0.4854 (2)	0.0539 (12)	
H23A	0.3144	0.0027	0.5077	0.081*	
H23B	0.2265	−0.0832	0.4486	0.081*	
H23C	0.1769	−0.0502	0.5224	0.081*	
C32	−0.0494 (4)	0.3293 (5)	0.3199 (2)	0.0556 (12)	
H32A	−0.0207	0.3982	0.3532	0.083*	
H32B	−0.087	0.2625	0.3457	0.083*	
H32C	−0.1158	0.3599	0.2803	0.083*	
C33	0.1154 (5)	0.4007 (4)	0.2276 (2)	0.0583 (13)	
H33A	0.191	0.3842	0.2039	0.087*	
H33B	0.132	0.4759	0.2576	0.087*	
H33C	0.0372	0.4134	0.1909	0.087*	
C42	0.2707 (6)	0.6430 (4)	0.3906 (3)	0.0814 (19)	
H42A	0.2891	0.6265	0.3421	0.122*	
H42B	0.3193	0.7174	0.4105	0.122*	
H42C	0.1766	0.6575	0.3881	0.122*	
C43	0.2747 (6)	0.5653 (6)	0.5310 (3)	0.094 (2)	
H43A	0.2969	0.5016	0.5686	0.141*	
H43B	0.1803	0.5815	0.5234	0.141*	
H43C	0.3223	0.6428	0.546	0.141*	
Ru1	0.26756 (3)	0.23282 (3)	0.374946 (14)	0.02744 (9)	
Cl1	0.44805 (10)	0.21704 (9)	0.48035 (5)	0.0419 (2)	
Cl2	0.41776 (11)	0.35631 (11)	0.31431 (6)	0.0504 (3)	
S11	0.32820 (10)	0.05696 (9)	0.31955 (5)	0.0368 (2)	
S21	0.14273 (9)	0.12074 (9)	0.44360 (5)	0.0336 (2)	
S31	0.08979 (10)	0.26855 (9)	0.28370 (5)	0.0367 (2)	
S41	0.32033 (11)	0.50995 (9)	0.44793 (6)	0.0416 (3)	

### Atomic displacement parameters (Å^2^)

**Table:**

	*U*^11^	*U*^22^	*U*^33^	*U*^12^	*U*^13^	*U*^23^
O11	0.069 (2)	0.0322 (15)	0.0534 (18)	−0.0047 (15)	−0.0011 (16)	−0.0051 (13)
O21	0.0335 (15)	0.0581 (18)	0.0453 (16)	−0.0134 (13)	−0.0011 (13)	0.0070 (14)
O31	0.0523 (18)	0.0505 (18)	0.0508 (18)	0.0058 (15)	−0.0175 (15)	−0.0114 (14)
O41	0.0403 (15)	0.0342 (14)	0.0439 (16)	−0.0031 (12)	0.0024 (13)	−0.0058 (12)
C12	0.046 (3)	0.073 (3)	0.056 (3)	0.024 (2)	−0.007 (2)	−0.014 (2)
C13	0.072 (3)	0.071 (3)	0.027 (2)	0.020 (3)	0.009 (2)	−0.005 (2)
C22	0.063 (3)	0.070 (3)	0.037 (2)	−0.018 (2)	0.019 (2)	−0.010 (2)
C23	0.053 (3)	0.051 (3)	0.056 (3)	−0.001 (2)	0.005 (2)	0.023 (2)
C32	0.041 (2)	0.071 (3)	0.052 (3)	0.021 (2)	−0.002 (2)	0.001 (2)
C33	0.073 (3)	0.058 (3)	0.041 (2)	0.009 (3)	0.001 (2)	0.020 (2)
C42	0.101 (4)	0.033 (2)	0.096 (4)	−0.009 (3)	−0.023 (4)	0.012 (3)
C43	0.129 (6)	0.099 (5)	0.059 (3)	−0.055 (4)	0.029 (4)	−0.043 (3)
Ru1	0.02955 (16)	0.02723 (15)	0.02414 (15)	−0.00181 (12)	0.00061 (11)	0.00173 (12)
Cl1	0.0378 (5)	0.0465 (6)	0.0364 (5)	−0.0057 (4)	−0.0081 (4)	0.0024 (4)
Cl2	0.0538 (7)	0.0539 (6)	0.0456 (6)	−0.0136 (5)	0.0145 (5)	0.0090 (5)
S11	0.0389 (5)	0.0376 (5)	0.0310 (5)	0.0053 (4)	−0.0018 (4)	−0.0038 (4)
S21	0.0341 (5)	0.0369 (5)	0.0285 (5)	−0.0058 (4)	0.0010 (4)	0.0031 (4)
S31	0.0396 (5)	0.0357 (5)	0.0311 (5)	0.0054 (4)	−0.0045 (4)	0.0015 (4)
S41	0.0448 (6)	0.0339 (5)	0.0433 (6)	−0.0039 (4)	−0.0007 (5)	−0.0037 (4)

### Geometric parameters (Å, °)

**Table:**

O11—S11	1.473 (3)	C32—S31	1.779 (4)
O21—S21	1.473 (3)	C32—H32A	0.96
O31—S31	1.484 (3)	C32—H32B	0.96
O41—S41	1.538 (3)	C32—H32C	0.96
O41—Ru1	2.158 (3)	C33—S31	1.772 (4)
C12—S11	1.784 (4)	C33—H33A	0.96
C12—H12A	0.96	C33—H33B	0.96
C12—H12B	0.96	C33—H33C	0.96
C12—H12C	0.96	C42—S41	1.769 (4)
C13—S11	1.781 (4)	C42—H42A	0.96
C13—H13A	0.96	C42—H42B	0.96
C13—H13B	0.96	C42—H42C	0.96
C13—H13C	0.96	C43—S41	1.770 (5)
C22—S21	1.792 (4)	C43—H43A	0.96
C22—H22A	0.96	C43—H43B	0.96
C22—H22B	0.96	C43—H43C	0.96
C22—H22C	0.96	Ru1—S11	2.2404 (10)
C23—S21	1.786 (4)	Ru1—S21	2.2640 (10)
C23—H23A	0.96	Ru1—S31	2.2780 (10)
C23—H23B	0.96	Ru1—Cl2	2.4126 (11)
C23—H23C	0.96	Ru1—Cl1	2.4380 (10)
			
S41—O41—Ru1	119.18 (15)	S41—C43—H43A	109.5
S11—C12—H12A	109.5	S41—C43—H43B	109.5
S11—C12—H12B	109.5	H43A—C43—H43B	109.5
H12A—C12—H12B	109.5	S41—C43—H43C	109.5
S11—C12—H12C	109.5	H43A—C43—H43C	109.5
H12A—C12—H12C	109.5	H43B—C43—H43C	109.5
H12B—C12—H12C	109.5	O41—Ru1—S11	176.60 (8)
S11—C13—H13A	109.5	O41—Ru1—S21	90.19 (8)
S11—C13—H13B	109.5	S11—Ru1—S21	93.00 (4)
H13A—C13—H13B	109.5	O41—Ru1—S31	86.21 (7)
S11—C13—H13C	109.5	S11—Ru1—S31	92.46 (3)
H13A—C13—H13C	109.5	S21—Ru1—S31	92.82 (4)
H13B—C13—H13C	109.5	O41—Ru1—Cl2	87.83 (8)
S21—C22—H22A	109.5	S11—Ru1—Cl2	89.12 (4)
S21—C22—H22B	109.5	S21—Ru1—Cl2	173.64 (4)
H22A—C22—H22B	109.5	S31—Ru1—Cl2	93.07 (4)
S21—C22—H22C	109.5	O41—Ru1—Cl1	86.78 (7)
H22A—C22—H22C	109.5	S11—Ru1—Cl1	94.59 (4)
H22B—C22—H22C	109.5	S21—Ru1—Cl1	86.31 (3)
S21—C23—H23A	109.5	S31—Ru1—Cl1	172.93 (4)
S21—C23—H23B	109.5	Cl2—Ru1—Cl1	87.54 (4)
H23A—C23—H23B	109.5	O11—S11—C13	106.3 (2)
S21—C23—H23C	109.5	O11—S11—C12	106.7 (2)
H23A—C23—H23C	109.5	C13—S11—C12	99.4 (2)
H23B—C23—H23C	109.5	O11—S11—Ru1	119.14 (14)
S31—C32—H32A	109.5	C13—S11—Ru1	112.43 (15)
S31—C32—H32B	109.5	C12—S11—Ru1	110.94 (15)
H32A—C32—H32B	109.5	O21—S21—C23	106.15 (19)
S31—C32—H32C	109.5	O21—S21—C22	106.02 (19)
H32A—C32—H32C	109.5	C23—S21—C22	99.8 (2)
H32B—C32—H32C	109.5	O21—S21—Ru1	119.85 (12)
S31—C33—H33A	109.5	C23—S21—Ru1	113.59 (15)
S31—C33—H33B	109.5	C22—S21—Ru1	109.35 (15)
H33A—C33—H33B	109.5	O31—S31—C33	106.36 (19)
S31—C33—H33C	109.5	O31—S31—C32	107.3 (2)
H33A—C33—H33C	109.5	C33—S31—C32	98.3 (2)
H33B—C33—H33C	109.5	O31—S31—Ru1	119.07 (12)
S41—C42—H42A	109.5	C33—S31—Ru1	112.47 (16)
S41—C42—H42B	109.5	C32—S31—Ru1	111.21 (14)
H42A—C42—H42B	109.5	O41—S41—C42	104.3 (2)
S41—C42—H42C	109.5	O41—S41—C43	101.9 (2)
H42A—C42—H42C	109.5	C42—S41—C43	99.7 (3)
H42B—C42—H42C	109.5		
			
S41—O41—Ru1—S21	135.85 (16)	S11—Ru1—S21—C23	45.78 (17)
S41—O41—Ru1—S31	−131.33 (16)	S31—Ru1—S21—C23	138.39 (17)
S41—O41—Ru1—Cl2	−38.11 (16)	Cl1—Ru1—S21—C23	−48.64 (17)
S41—O41—Ru1—Cl1	49.55 (16)	O41—Ru1—S21—C22	−24.90 (19)
S21—Ru1—S11—O11	15.75 (14)	S11—Ru1—S21—C22	156.27 (17)
S31—Ru1—S11—O11	−77.21 (14)	S31—Ru1—S21—C22	−111.12 (17)
Cl2—Ru1—S11—O11	−170.25 (14)	Cl1—Ru1—S21—C22	61.86 (17)
Cl1—Ru1—S11—O11	102.29 (14)	O41—Ru1—S31—O31	−165.41 (17)
S21—Ru1—S11—C13	141.01 (18)	S11—Ru1—S31—O31	17.72 (16)
S31—Ru1—S11—C13	48.05 (18)	S21—Ru1—S31—O31	−75.41 (16)
Cl2—Ru1—S11—C13	−44.99 (18)	Cl2—Ru1—S31—O31	106.97 (16)
Cl1—Ru1—S11—C13	−132.46 (18)	O41—Ru1—S31—C33	69.24 (19)
S21—Ru1—S11—C12	−108.64 (18)	S11—Ru1—S31—C33	−107.63 (18)
S31—Ru1—S11—C12	158.40 (18)	S21—Ru1—S31—C33	159.24 (17)
Cl2—Ru1—S11—C12	65.36 (18)	Cl2—Ru1—S31—C33	−18.38 (18)
Cl1—Ru1—S11—C12	−22.10 (18)	O41—Ru1—S31—C32	−39.94 (19)
O41—Ru1—S21—O21	97.69 (16)	S11—Ru1—S31—C32	143.19 (18)
S11—Ru1—S21—O21	−81.13 (15)	S21—Ru1—S31—C32	50.06 (18)
S31—Ru1—S21—O21	11.48 (15)	Cl2—Ru1—S31—C32	−127.56 (18)
Cl1—Ru1—S21—O21	−175.54 (15)	Ru1—O41—S41—C42	116.2 (2)
O41—Ru1—S21—C23	−135.40 (18)	Ru1—O41—S41—C43	−140.4 (3)
